# Poly(Ionic Liquid) Based Chemosensors for Detection of Basic Amino Acids in Aqueous Medium

**DOI:** 10.3389/fchem.2017.00069

**Published:** 2017-09-26

**Authors:** Xinjuan Li, Kai Wang, Nana Ma, Xianbin Jia

**Affiliations:** Key Laboratory of Green Chemical Media and Reactions, School of Chemistry and Chemical Engineering, State Education Ministry of China, Henan Normal University, Xinxiang, China

**Keywords:** poly-ionic liquid, RAFT polymerization, naked-eye sensor, amino acids, water

## Abstract

Naked-eye detection of amino acids (AA) in water is of great significance in the field of bioanalytical applications. Herein, polymerized ionic liquids (PILs) with controlled chain length structures were synthesized via reversible addition–fragmentation chain-transfer (RAFT) polymerization and post-quaternization approach. The AA recognition performance of PILs with different alkyl chain lengths and molecular weights was evaluated by naked-eye color change and ultraviolet-visible (UV–vis) spectral studies. These PILs were successfully used for highly sensitive and selective detection of Arg, Lys, and His in water. The recognition performance was improved effectively with increased molecular weight of PILs. The biosensitivity of the PILs in water was strongly dependent on their aggregation effect and polarization effect. Highly sensitive and selective detection of AA was successfully accomplished by introducing positively charged pyridinium moieties and controlled RAFT radical polymerization.

## Introduction

Amino acids (AA) play an important key role in many physiological processes (Mohr et al., 1998; Shahrokhian, 2010). With increasing attention paid to human health, including diagnosis and treatment of diseases, scientists have devoted a lot of energy in exploring new methods for amino acid analysis (Vychytil et al., 2003; Zhou and Yoon, 2012). The traditional detection method mainly comprises the introduction of some electrophilic groups and structures like aldehyde that react with amine group (Xu et al., 2017). However, the prevalent problem is not only the solubility of AA in water but also the occurrence of weak intermolecular interaction with recognition receptors in water (Riikka et al., 2014; Samantha and Francesca, 2014). Hence, it is urgently needed to develop new amino acid sensors that are not only highly sensitive but also capable of molecular recognition in aqueous system that will aid in the enhancement of bioanalytical applications (Ooyama et al., 2013).

In recent years, molecule-based ion sensors are getting considerable attention because of their interaction with Lewis basic substrates in water, resulting in marked color and/or luminescence changes (Li et al., 2013; Ding et al., 2015; Wan et al., 2017). Gold nanoparticles modified with *p*-sulfonato-1, 3-dialkoxycalix[4]arene thiol have been reported to probe AA in aqueous solution (Patel and Menon, 2009). Phosphonate cavitands have been known as powerful receptors that possess molecular recognition properties toward AA (Roberta et al., 2016). In the process of amino acid detection, the calix[n]arenes have aroused many researchers' concern and interest. However, the traditional sensors have some disadvantages requiring time-consuming design and more synthetic steps (Sasaki et al., 2002). Recently, water-soluble conjugated polymers with charged groups as novel biosensors have shown tremendous potential for protein recognition (Huang et al., 2005; Chen et al., 2009, 2011). All the analytical processes were performed mainly through electron transfer (ET) and electrostatic interaction (Vilkanauskyte et al., 2002). However, such polymer sensor was limited to the conjugated structure with ET center (Tan et al., 2012), whereas no other work is reported using polymer without ET center for the biosensing applications (Zhou et al., 2011).

Polymer ionic liquid (PIL) is the polymer that contains repeating units of ionic liquid. Polymerized ionic liquids (PILs) possess unique features that belong to both polymer and counter intrinsic properties of IL with anion and cation (Zhang et al., 2013; Mahsa et al., 2017). In recent years, PILs have been widely applied in organic dispersant, nano composite materials, electrochemical, adsorbent, and separation techniques (Reeca et al., 2006; Mecerreyes, 2011; Li et al., 2017). Although PILs have extensive application values, their application as molecular probes for detection of AA in water has not been reported yet.

In this paper, we introduce a kind of PILs with well-controlled architectures via reversible addition-fragmentation chain-transfer polymerization (RAFT polymerization) and post-quaternization approach (Yuan et al., 2011; Mori et al., 2012). The AA recognition performance of PILs with different alkyl chain lengths and molecular weights was evaluated by ultraviolet-visible (UV–vis) spectral studies and color changes in ethanol and in pure water. The experimental results and theoretical analysis showed that PILs detected amino acid in ethanol by the strong ionic interactions and hydrogen bonding interactions. These PILs were successfully used for highly sensitive and selective detection of Arg, Lys, and His in water, whose recognition was dependent on the aggregation effect and electrostatic of PILs and basic AA. To the best of our knowledge, no work has been reported so far on the polymeric ionic liquid that can detect AA in water in highly sensitive manner without any special recognition groups.

## Materials and methods

### Materials

All the AA and alkyl halides were purchased from Aladdin and AA standards are of 98–99% purity. 4-Vinylpyridine (4-VP) and N, N-Dimethylformamide (DMF) were purified by distillation after pressure. The chain transfer agent cumyl dithiobenzoate (CDB) was synthesized according to literature method (Li et al., 2015). The initiator 2, 2′-azobis (2-methylpropionitrile) (AIBN) was purchased from Sigma-Aldrich and purified by recrystallization in ethanol before use. The other reagents were used as received.

### Instruments

A Cary 1E UV–vis spectrophotometer was used to measure absorption spectra at 25°C. ^1^H NMR spectral analysis was carried out using Bruker AV-400 NMR spectrometer. FT-IR spectra were performed on a Nicolet NEXUS Fourier transform infrared spectrometer. Dynamic light scattering (DLS) was performed on Nano Particle Analyzer (Zetasizer Nano ZS90, Malvern Instruments). Gel permeation chromatography (GPC) was performed on a Waters 1515 apparatus.

### Preparation and characterization of PILs

4-VP (3.85 mmol), AIBN (3.1 mg, 0.019 mmol), CDB (20.7 mg, 0.076 mmol), and DMF (5 mL) were added into a 10 mL round-bottom flask. A clear solution was obtained after stirring for 10 min. After five freeze pump thaw cycles for degassing in order to remove the oxygen from the system, the flask was sealed then and placed at 65°C for 48 h. Thereafter, the product was subsided in ether, centrifuged, and washed with ether to remove the unreacted 4-VP monomer. The polymer product, P4VP_1_, obtained was dried in a vacuum at 40°C.

P4VP_2_ was prepared by using the similar method: 4-VP (7.61 mmol), AIBN (3.1 mg, 0.019 mmol), CDB (20.7 mg, 0.076 mmol), and DMF (5 mL) were added into a 10 mL round-bottom flask. The sample is treated and purified in a same way as P4VP_1_.

P4VP_1_ or P4VP_2_ (0.1 g, 0.95 mmol) was added into 3 mL chloroform, followed by the addition of bromoethane (9.5 mmol). The reaction mixture was heated at 60°C for 48 h and then kept for cooling at room temperature. The solid was separated out and washed with chloroform for five times to remove the unreacted P4VP. The resulting solid was filtered and vacuum-dried at 40°C to produce the P4VP_1_-Br_2_ and P4VP_2_-Br_2_. P4VP_1_-Br_4_ and P4VP_1_-Br_8_ were synthesized by the reaction of P4VP_1_ with n-butyl bromide or n-octyl bromide, respectively.

### FTIR characterization

FT-IR spectra were recorded on a Nicolet NEXUS Fourier transform infrared spectrometer, and the samples were prepared as follows: the PIL sample was dissolved in ethanol and then dripped on a potassium bromide sheet to make it dry perfectly. P4VP_1_-Br_2_ solution (4.3 mM in ethanol) with addition of 2 × 10^−4^ M L-Arg was also prepared using the same method. The spectra were performed in a Biorad FTS 165 spectrophotometer with a resolution of 4 m^−1^ (32 scans).

### DLS measurement

The average hydrodynamic sizes of PIL in water were determined by DLS using a Nano Particle Analyzer with a He Ne laser and 90° collecting optics operating at λ = 660 nm at 25°C. The samples were prepared as follows: P4VP_1_-Br_2_ solution (4.3 mM in water) with the addition or no addition of 2 × 10^−4^ M L-Arg. The solution was filtered using a 0.22 μm filters.

### GPC measurement

The molecular weights and molecular weight distribution (PDI = M_w_/M_n_) of the synthesized polymer samples (5 mg/mL DMF solution) were determined by gel permeation chromatography (GPC) equipped with a Waters 1515 apparatus and a PD2020 light scattering detector, using DMF as eluent. The flow rate was 1.0 mL.min^−1^, and polystyrene samples were used as standards.

### General UV–vis spectral measurements

In the titration part, PIL solution (4.3 mM) was prepared in ethanol or water. UV–vis spectra were also obtained in ethanol or water solution. Other AA (0–2 × 10^−4^ M) and chemicals were prepared in deionized water or ethanol. The limit of detection (LOD) for Arg is calculated according to a signal-to-noise ratio of 3 (ΔI = I–I_0_), where I and I_0_ are the wavelength intensities of the PIL in the presence and absence of Arg, respectively (Lu et al., 2017).

## Results and discussion

### Synthesis and characterization of PILs

**Figure 1:**
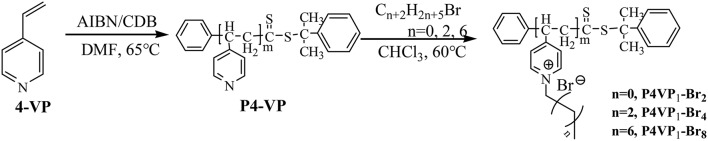
The synthesized method of polymer ionic liquid.

The synthetic procedure of PILs with different alkyl chain lengths was outlined in Figure 1. The neutral polymers P4VPs with different molecular weights were obtained through RAFT polymerization, azo-diisobutyronitrile (AIBN) as initiator agent, and CDB as RAFT chain transfer agent. GPC analyses demonstrated that P4VPs with narrow molecular weight distribution were successfully obtained (Table 1). In the two cases, the molecular weights from GPC are far smaller than the values obtained from ^1^H NMR (Figure S1). This may be attributed to the different aggregates in solution or the deviation from the polystyrene samples (Schilli et al., 2004). The neutral polymers P4VP_1_ and P4VP_2_ were converted to corresponding P4VP_1_-Br_2_ and P4VP_2_-Br_2_, which were achieved by stirring the polymer with bromoethane in CHCl_3_ at 60°C for 2 d. Such a post-quaternization approach is highly valuable regarding the easy purification and further characterization of neutral polymer products. Meanwhile, P4VP_1_-Br_4_ and P4VP_1_-Br_8_ were also obtained by reacting the P4VP_1_ with n-butyl bromide and n-octyl bromide, respectively.

**Table 1:** Polymer molecular weight data of P4VP.

**Sample^a^**	**Monomer/CDB/AIBN (mole ratio)**	**M_n_, _Gpc_^a^**	**PDI**	**M, n_NMR_^b^**
P4VP_1_	202/4/1	1991	1.41	8672
P4VP_2_	400/4/1	5301	1.29	21272

a *GPC was used to measure molecular weights of the polymers*.

b *Determined by ^1^H NMR spectroscopic analysis in CDCl_3_, by comparing the proton peak intensity of P4VP (δ 8.2–8.6 ppm, 2H) and the RAFT end-group (δ 6.8–7.9 ppm, 5H)*.

### Molecular recognition ability of PIL for detecting amino acid in ethanol

**Figure 2:**
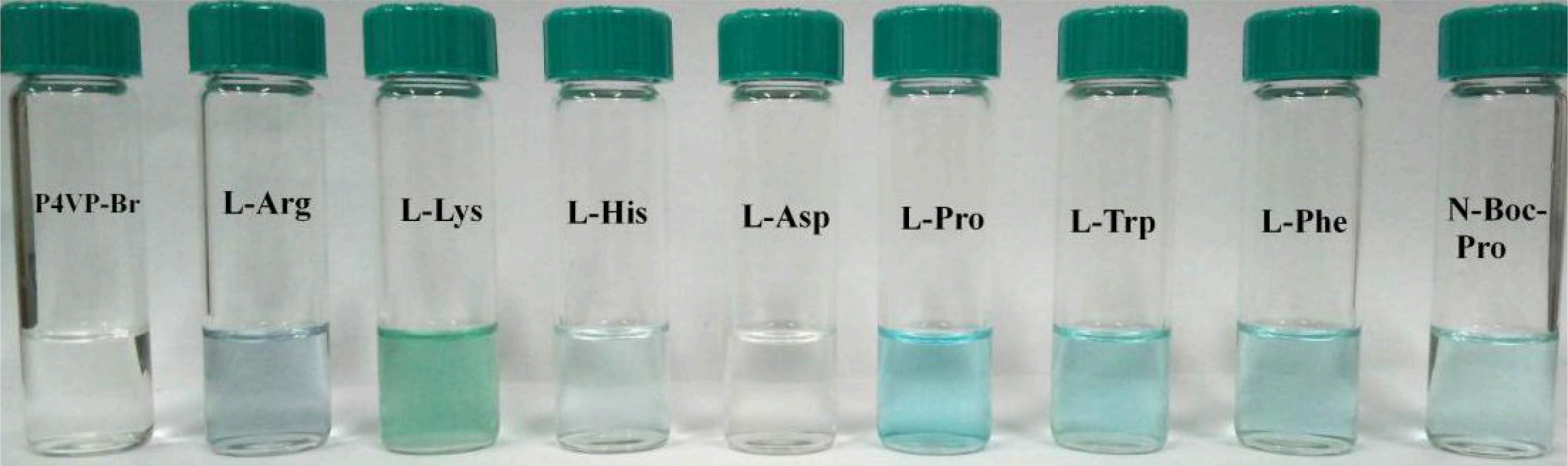
Photographic images of P4VP_1_-Br_2_ ethanol solutions containing different amino acids.

**Figure 3:**
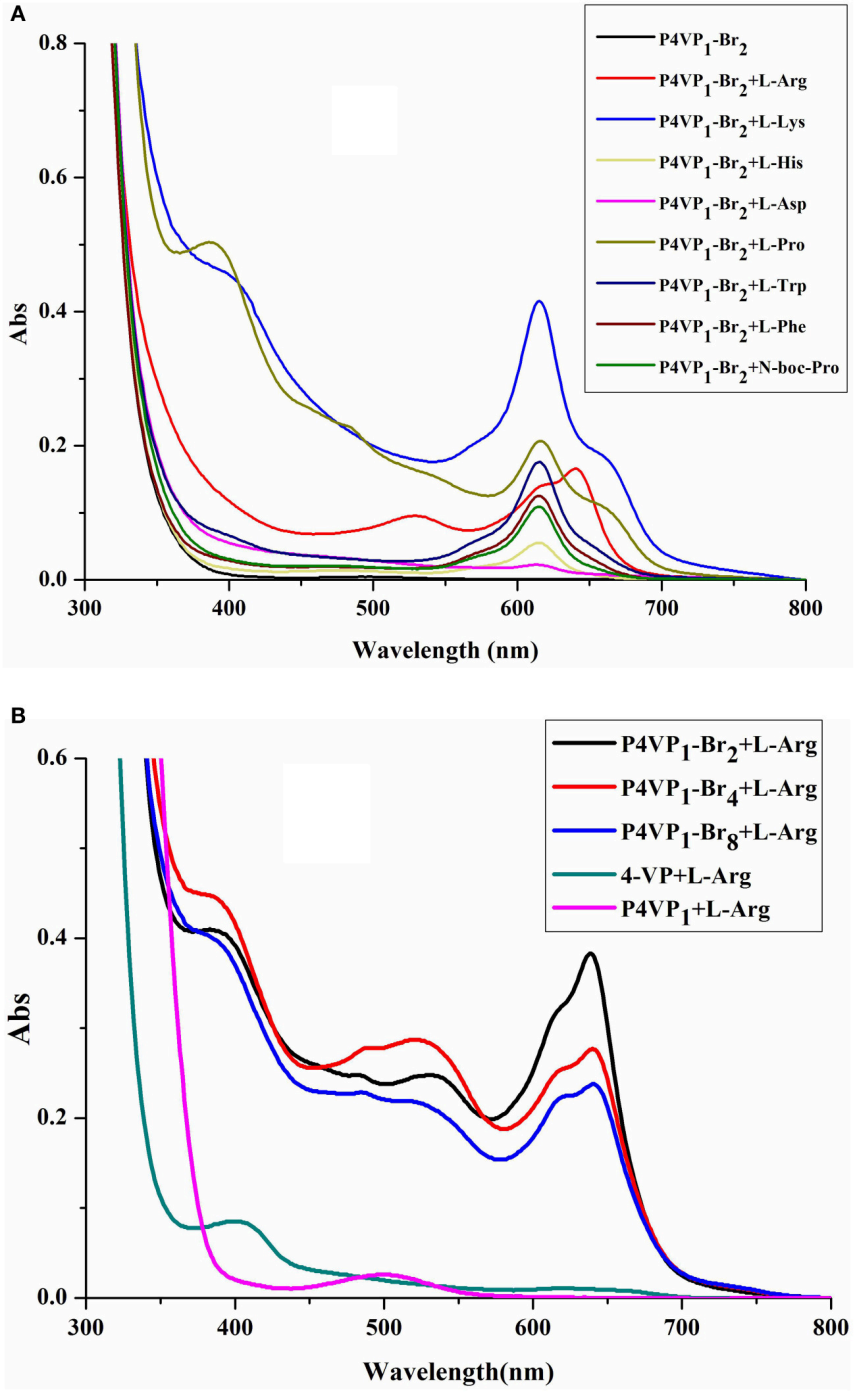
UV-visible spectra of 4.3 mM P4VP_1_-Br_2_ responding to 1 × 10^−4^ M amino acids in ethanol solutions **(A)**, and different PILs ethanol solutions of 4.3 mM P4VP_1_-Br_2_ with the addition of 1 × 10^−4^ M L-Arg **(B)**.

In order to evaluate the recognition ability of PIL, 8 different AA (2 × 10^−4^ M) were added to P4VP_1_-Br_2_ ethanol solutions (4.3 mM). A few seconds later, the colorless solutions containing Arg, Lys, His, Pro, Trp, Phe, and N-Boc-Pro were found turning to blue color (Figure 2). At the same time, UV-vis spectral analysis showed the appearance of a new absorption peak at 600–650 nm on the addition of these AA (Figure 3A). However, the color or adsorption spectrum of P4VP_1_-Br_2_ was found to have no effect on the addition of acidic AA (e.g., Asp). In addition, basic AA (Arg and Lys) showed more obvious recognition than the neutral AA and acidic AA, which are the evidences of occurrence of electrostatic interaction between the PIL and AA with carboxyl groups. The recognition performance for N-Boc proline suggests that the key factor for recognition is the presence of carboxyl groups. But the stable identification for AA depends on the presence of amino groups because the color and UV-vis spectral changes of the ethanol solution for P4VP_1_-Br_2_ and N-Boc Proline disappeared after placing 24 h at room temperature (Figures S2, S3).

Control experiments of 4-VP monomer and P4VPs with the addition of Arg, Lys, or His showed no peaks in the range of 600–650 nm and also no color change was observed. This demonstrated that the recognition performance of PIL might be due to its aggregated and polarized nature (Wang et al., 2011).

In addition, we have also studied the effect of different alkyl chain lengths on the recognition performance of PILs. There were similar changes in the UV-vis spectra of P4VP_1_-Br_4_ and P4VP_1_-Br_8_ induced by the addition of Arg (Figure 3B). It was noticed that with increase in the alkyl chain length, the recognition performance decreased. P4VP_1_-Br_2_ with the shortest alkyl chain length was found to exhibit the best molecular recognition performance. For pyrrolidinium ionic liquids, the length of alkyl chain on the cation was observed to have a significant influence on their physicochemical properties (such as the polarity and conductivity). The decreased recognition performance of P4VP_1_-Br_4_ and P4VP_1_-Br_8_ might be due to the steric effects causing the increasing distance between the PIL and amino acid (Lee and Prausnitz, 2010).

To understand the geometry of the PIL-amino acid 1:2 complex, the density functional theory (DFT) calculations were carried out using Gaussian 09 package (Rohit et al., 2007; Rega et al., 2009; Yan et al., 2012). The B3LYP/6-31+G(d)-optimized structures for the complexes of receptor with Arg are represented in Figure S4. The theoretical results indicated that the strength of interactions among the ionic interactions and the hydrogen bonding interactions between different polymer chains and amino acid groups might be the important factor responsible for better recognition performance of PILs than their corresponding monomer counterparts.

**Figure 4:**
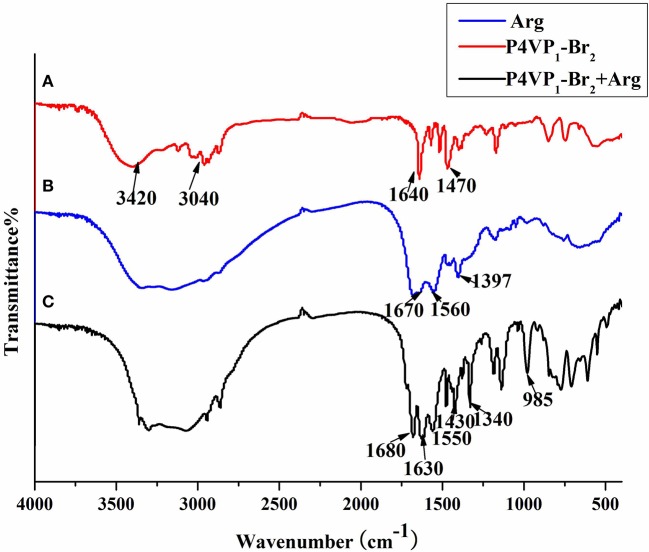
FTIR of P4VP_1_-Br_2_ **(A)**, L-Arg **(B)**, and P4VP_1_-Br_2_ after combining with L-Arg **(C)**.

Furthermore, the FTIR spectra of P4VP_1_-Br_2_ before and after amino acid recognition are presented in Figure 4. There were some common peaks appeared at around 3,040, 1,640, and 1,470 cm^−1^, which were the typical characteristic peaks of P4VP_1_-Br_2_. After combination with amino acid in solution, the new peaks at 1,630 and 1,340 cm^−1^ were obtained that attributed to ionic bond interactions between the two moieties (Zhou et al., 2012). After PIL reacted with the carboxylic acid group of amino acid, a new structure was formed that marked the significant recognition performance of PIL.

### Molecular recognition ability of PIL for detecting amino acid in pure water

**Figure 5:**
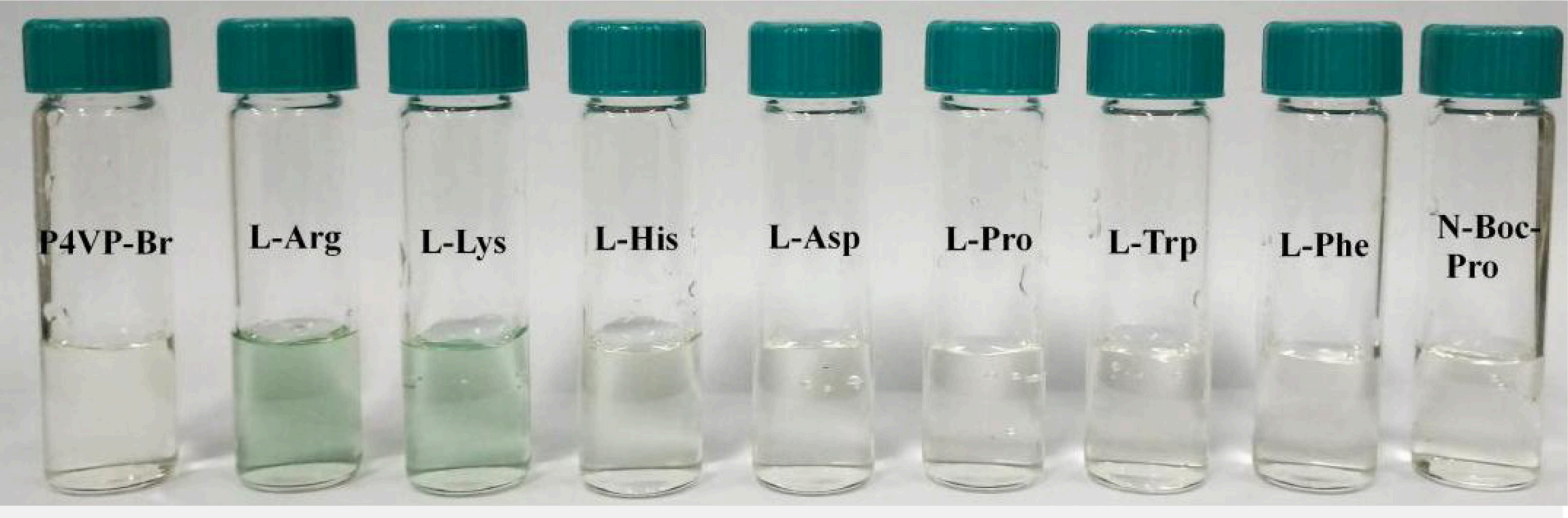
Photographic images of P4VP_1_-Br_2_ water solutions containing different amino acids.

**Figure 6:**
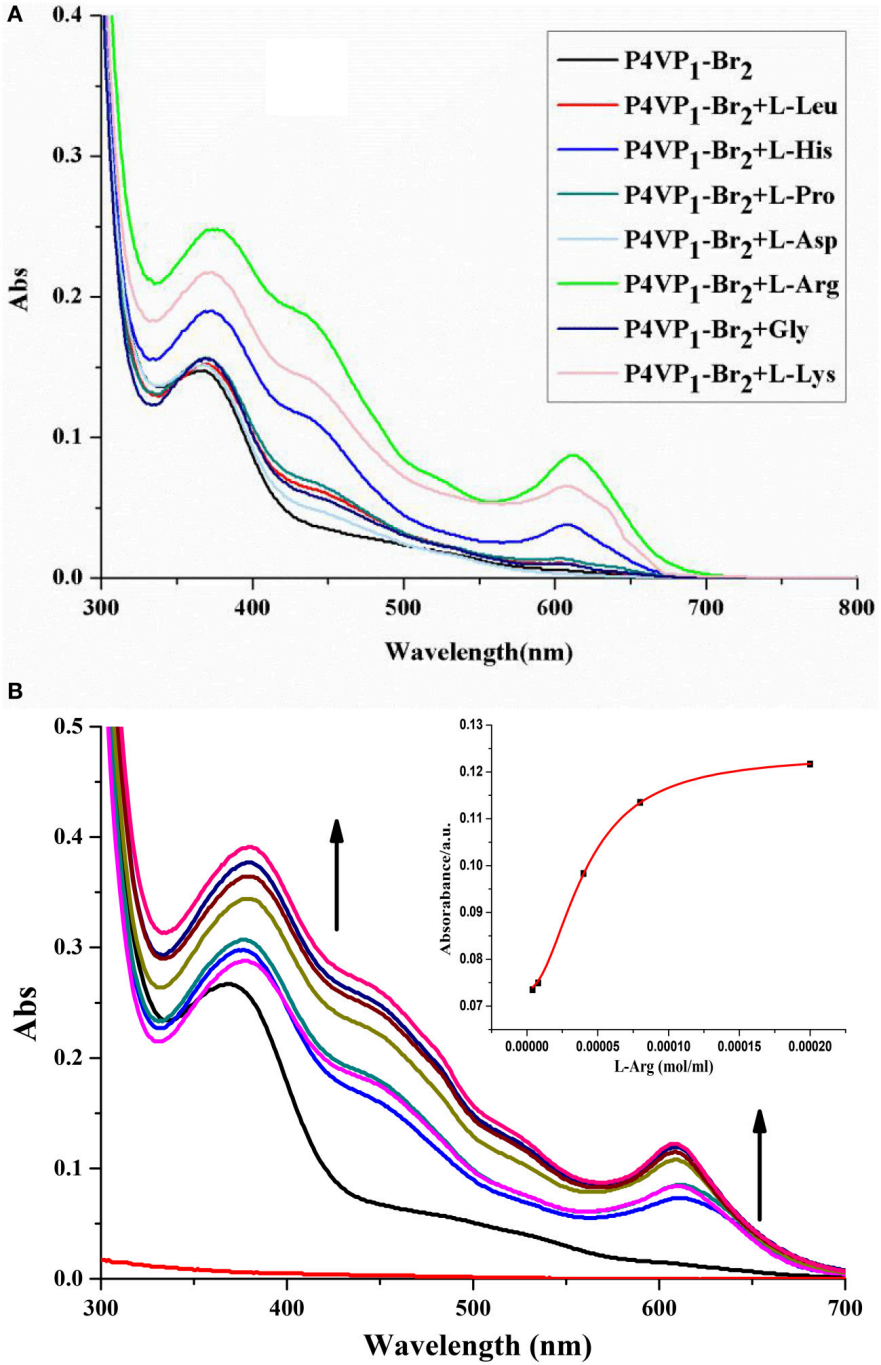
UV-vis spectra of P4VP_1_-Br_2_ (4.3 mM) in water in the presence of 2 × 10^−4^ M different amine acids **(A)** and Evolution of UV-vis spectrum of P4VP_1_-Br_2_ (4.3 M) on addition of L-Arg = 0–2 × 10^−4^ M in water. The inset showed the changes of absorbance at 610 nm against the added L-Arg **(B)**.

**Figure 7:**
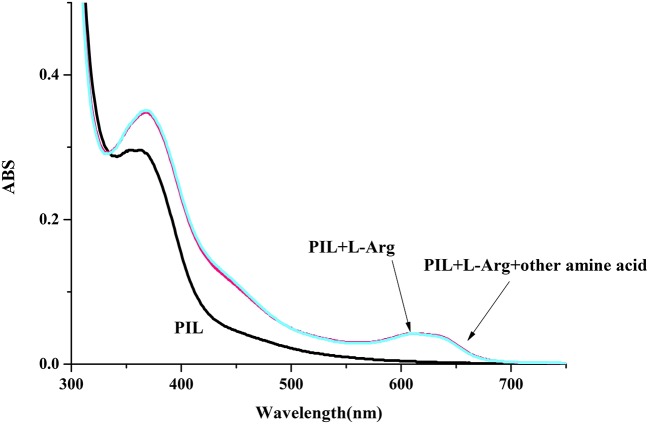
UV-vis spectrum of P4VP_1_-Br_2_ (4.3 mM) on the addition of L-Arg (1 × 10^−4^ M) in water (the concentration of the other amine acid is 1 × 10^−4^ M).

Limited by the insolubility of other PILs in water, we investigated the molecular recognition ability of P4VP_1_-Br_2_ in pure water. Different AA (2 × 10^−4^ M) were added into P4VP_1_-Br_2_ aqueous solution (4.3 mM). After a short period, the colorless solutions containing Arg, Lys, and His were found turning green that could be readily visualized by the naked eyes (Figure 5). However, the P4VP_1_-Br_2_ solution containing the other AA showed no color or adsorption spectral changes, which indicated that the P4VP_1_-Br_2_ responded selectively to Lys, Arg, and His. In Figure 6, it is clear that there is a significant change in absorbance intensity at 610 nm upon addition of Lys, Arg, and His. With the addition of Arg (0–2 × 10^−4^ M), absorption peak at 375 nm showed great increase in intensity along with the appearance of a new peak at 610 nm in UV–vis spectra. Interestingly, the peak at 375 nm, which was assigned to π-π stacking of PIL, increased with the addition of Arg. This result demonstrated that the interactions between PIL and Arg might be due to the aggregation effect. It is clear that the absorbance intensity at around 610 nm showed a significant change with increased concentration of these three AA. Binding constant of 6.1 × 10^8^ for 1:2 binding of PIL:Arg was obtained. The binding constants for Lys and His were calculated as 5.1 × 10^8^ and 2.9 × 10^7^, and the linear fitting constants were found as 0.999, 0.997, and 0.991 for Arg, Lys, and His, respectively (Table 2). Particularly, the addition of other AA (Phe, Proline, Asp, and N-Boc-Pro) had no effect on the color or UV–vis adsorption spectrum for P4VP_1_-Br_2_ and Arg (Figure 7), which showed the selective recognition ability of PIL for basic AA. In water solution, on one hand, due to high polarity of water, part of the hydrogen bond was damaged and the hydrogen bonding interaction between AA and PIL was greatly reduced. On the other hand, the interaction of positive and negative charges promoted the predetermined selectivity of PILs for basic AA. In order to understand the crucial interactions involved in recognition, we also did the following experiments: a. P4VP_1_-Br_2_+Gnd.HCl (guanidine.HCl) in water and b. P4VP_1_-Br_2_+NH_4_Cl in water. However, no response was observed in the UV absorption and color changes in these experiments. The results indeed demonstrated the very high selectivity of the synthesized quaternary PIL, thereby highlighting the importance of amino acid functionality in addition to basic side chain group in the recognition.

**Table 2:** K_a_ and R obtained from the titration of PIL with the interacting Amino Acids (aa).

**PIL**	**aa**	**K_a(610nm)_**	**R**
P4VP_1_-Br_2_	Arg	6.1 × 10^8^	0.999
	Lys	5.1 × 10^8^	0.997
	His	2.9 × 10^7^	0.991
P4VP_2_-Br_2_	Arg	7.1 × 10^8^	0.993
	Lys	5.9 × 10^8^	0.997
	His	4.3 × 10^7^	0.996

Furthermore, we have studied the effect of molecular weights of polymer on the recognition performance. Interestingly, the recognition performance of P4VP_2_-Br_2_ with the same alkyl chain length but different molecular weights compared with P4VP_1_-Br_2_ for L-Arg appeared to increase in water. The binding constant for Arg was 7.1 × 10^8^, and the coefficient of the linear fit was 0.993 (Table 2). Here, the detection limit of Arg was 0.026 μM, which was close to the minimum detection limit for the determination of Arg among all the developed approaches as shown in Table 3. PIL as amino acid sensor shows the high sensitivity in water. P4VP_2_-Br_2_ also showed the better recognition performance for Lys and His than P4VP_1_-Br_2_. This might be due to increased aggregation phenomenon with the increase of molecular weight, and hence, recognition performance increased substantially. It is advantageous that the recognition performance of the polymer sensor can be simply regulated by varying the molecular weights accordingly.

**Table 3:** Detection limit of Arg by various detection methods.

**Detection method**	**Detection limit (μM)**	**References**
P4VP_2_-Br_2_	0.026	Present work
Fluorescent sensor	0.17	Cao et al., 2014
Phosphorescence sensor	2.3	He et al., 2014
Visual detection based on nanoparticle	0.016	Pu et al., 2013
Evanescent wave infrared chemical sensor	5	De orduña, 2001
Fluorescent sensor	2.05	Wei and Yang, 2007
Visual detection and Fluorescent sensor	0.034	Liu et al., 2017

**Figure 8:**
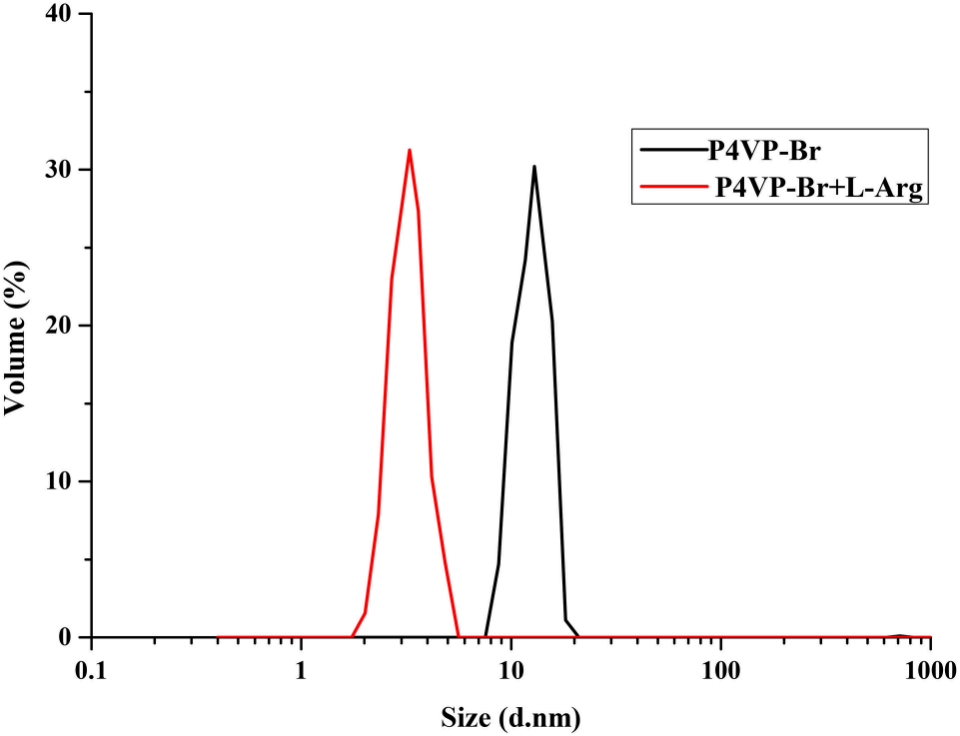
Dynamic light scattering of P4VP_1_-Br_2_ 4.3 mM before and after treatment with 2 × 10^−4^ M Arg.

Dynamic light scattering (DLS) experiments were used to measure the hydrodynamic diameters of P4VP_1_-Br_2_ in the presence of Arg (Figure 8). DLS results indicated that Arg-involved assembly structures comprise a much smaller volume in such complex structures. The polarity of cation of P4VP_1_-Br_2_ was found effective for the electrostatic interaction between polymer and the carboxyl group of Arg. It was noticed that Arg and PIL tend to aggregate and assemble more easily than the other AA with PIL (Patel and Menon, 2009). The further detailed analysis is currently under investigation.

## Conclusions

In summary, a water-soluble polymer ionic liquid (PIL) sensor is synthesized using RAFT polymerization and post-quaternization approach. The PIL acted as a highly efficient colorimetric sensor and selectively detected the basic AA in an aqueous environment. Further research proved that the recognition performance of PIL in ethanol depends on the strength of intermolecular electrostatic interactions and hydrogen-bond interactions between PIL and the AA. The selective recognition for basic AA in water could be owing to the aggregation effect and polarization effect. Deeper recognition mechanisms are being further studied. The new sensor was realized by simply introducing the positively charged pyridinium moieties and controlled RAFT radical polymerization that paved the new method for designing highly sensitive and highly selective biosensor for special AA recognition.

## Author contributions

XL and KW contributed to the experimental studies and manuscript preparation. NM contributed to the theory calculations. XJ contributed to the study design, manuscript revision, and final version approval.

### Conflict of interest statement

The authors declare that the research was conducted in the absence of any commercial or financial relationships that could be construed as a potential conflict of interest.
