# Supplementary material for: Poly(Ionic Liquid) Based Chemosensors for Detection of Basic Amino Acids in Aqueous Medium
Source: Front Chem. 2017 Sep 26;5:69. doi: 10.3389/fchem.2017.00069 (PMC5622980; doi:10.3389/fchem.2017.00069)
Supplement: Supplementary file 1 [file Presentation1.PDF]

Supporting Information

Poly(ionic liquid) based chemosensors for detection of basic amino acids in aqueous medium

Xinjuan Li[a,*], Kai Wang[a], Nana Ma[a], Xianbin Jia[a,*]

[a] School of Chemistry and Chemical Engineering, the Key Laboratory of Green Chemical Media and Reactions, State Education Ministry of China, Henan Normal University, Xinxiang 453007, P. R. China,

Correspondence to: Xinjuan Li(E-mail:) xinjuanli2009@163.com

Correspondence to: Xianbin Jia(E-mail:) axbjia@163.com

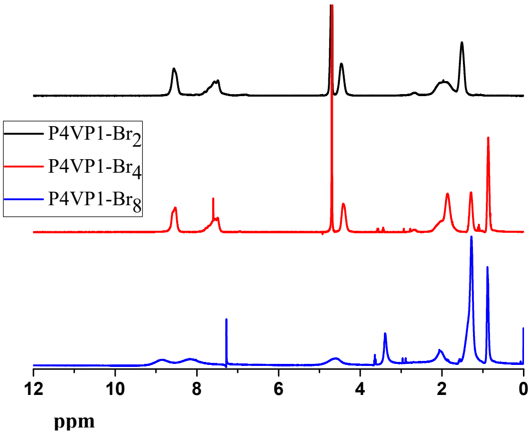

Fig. s1 ^{1}H NMR of PILs in $CDCl_3$.

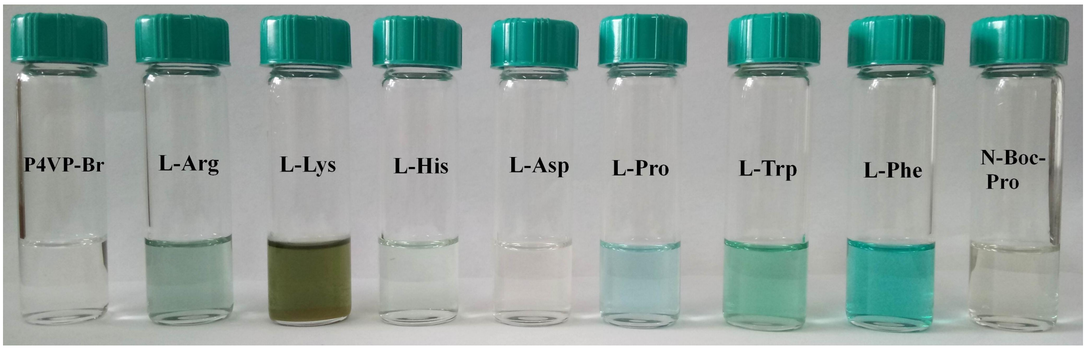

Fig. S2 Photographic images of $P4VP_1$-Br_2 ethanol solutions containing different amino acids after placing 24 h at room temperature.

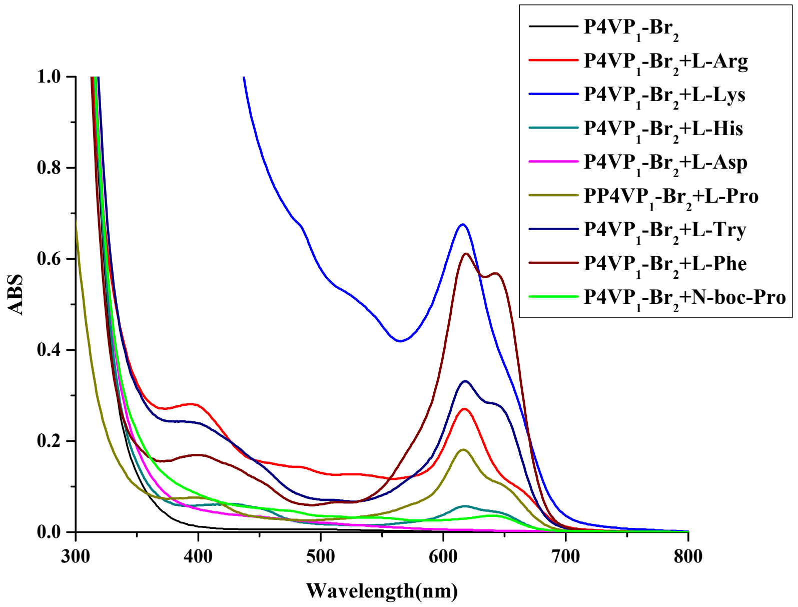

Fig. S3 UV-visible spectra of 4.3 mM $P4VP_1$-Br_2 responding to 1×10^{-4} M amino acids in ethanol solutions after placing 24 h at room temperature.

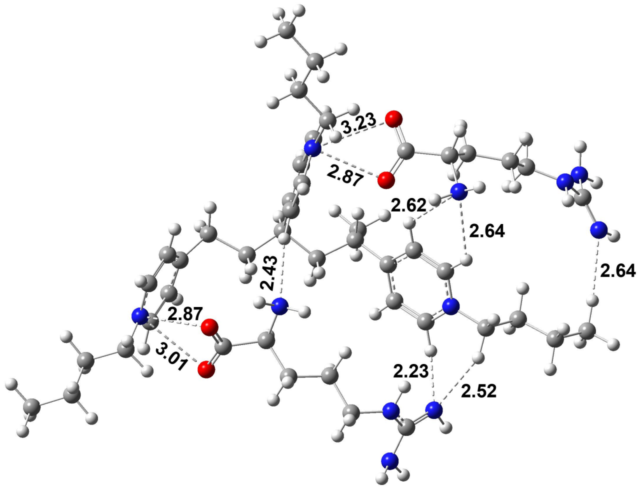

Fig S4. B3LYP/6-31+G(d)-predicted optimized geometry of complex of $P4VP_1$-Br_2 with Arg.

The B3LYP/6-31+G(d)-optimized structures for the complexes of receptor with Arg are represented in Fig. S4. As for Arg, carboxyl group participated in the formation of ion interaction with N^+ ($dO_{....}N$=2.87 A°), N^+ ($dO_{....}N$=3.01 A°)and ($dO_{....}N$=3.23 A°), and NH groups of Arg also formed hydrogen bonds with the protons on an adjacent polymer chain of PIL. These theoretical results indicated that the strength of interactions among the ionic interactions and the hydrogen bonding interactions between different polymer chains and amino acid groups, contributes to

the observation and might be one of the important factors responsible for better recognition performance of PILs than their corresponding monomer counterparts.
